# Evaluation of Commercial Anti-Listerial Products for Improvement of Food Safety in Ready-to-Eat Meat and Dairy Products

**DOI:** 10.3390/antibiotics12020414

**Published:** 2023-02-20

**Authors:** Pilar Colás-Medà, Inmaculada Viñas, Isabel Alegre

**Affiliations:** Postharvest Biology and Technology Unit, Department of Food Technology, Engineering and Science, University of Lleida, AGROTECNIO-CERCA Center, Av. Rovira Roure 191, 25198 Lleida, Spain

**Keywords:** *Listeria innocua*, cooked ham, dry-cured Spanish pork sausage, fresh cheese, LAB, probiotic bacteria, bacteriophage, *Leuconostoc carnosus*, *Pediococus acidilactici*, *L. rhamnosus* GG

## Abstract

In ready-to-eat products, such as cooked ham, fresh cheese, and *fuet* in which *Listeria monocytogenes* is a concern, the use of biopreservation techniques represents an additional hurdle to inhibit pathogen growth during storage. The objective of this study was to apply several biopreservation techniques in three different food matrices to reduce the growth of *Listeria innocua*, used as a surrogate of *L. monocytogenes*. Several lactic acid bacteria, the bacteriocin nisin, the bacteriophage PhageGuard Listex^TM^ P100, and the enzyme lysozyme were evaluated. Cooked ham treated with the bacteriophage PhageGuard Listex^TM^ at 0.5% or with the lactic acid bacteria SafePro^®^ B-SF-43 (25 g/100 kg) reduced *L. innocua* population to below the detection limit after 7 days of storage (4 °C plus modified atmosphere packaging). In fresh cheese, the application of PhageGuard Listex^TM^ at 0.2 and 0.5% reduced *L. innocua* counts by more than 3.4 logarithmic units after 6 days at 4 °C. In *fuet*, the 1.0% of PhageGuard Listex^TM^ reduced *L. innocua* population by 0.7 ± 0.2 logarithmic units in front of control with no significant differences to other evaluated biopreservative agents. The present results confirm that the application of biopreservation techniques was able to inhibit *L. innocua* in *fuet*, cooked ham, and fresh cheese, and suggest that the type of food matrix and its physicochemical characteristics influence the biopreservative efficacy.

## 1. Introduction

*Listeria monocytogenes* is a ubiquitous microorganism that can be found widely in the environment, but also in food processing plants where it can be protected from cleaning and disinfection operations due to its ability to produce biofilms. Therefore, *L. monocytogenes* can contaminate food at any point in its processing, from raw material production to household food handling. This microorganism causes invasive listeriosis which affects high-risk groups in the population: elderly people, newborns, immunocompromised people, and pregnant women.

Since *L. monocytogenes* is a quite resistant bacterium, able to grow at refrigeration temperatures (minimal growth temperature between −1.5 and 3.0 °C), at low pH and low water activity, ready-to-eat (RTE) foods with long shelf life are considered risky food products [1]. The main categories of RTE foods implicated in human listeriosis are ‘meat and meat products’, ‘fish and fish products’, and ‘milk and milk products’ [2]. Although the presence of *L. monocytogenes* in RTE foods is legislated in the European Union (EU) since 2006 as a food safety criterion (Commission Regulation (EC) 2073/2005), in the period from 2009–2013 a statistically significant increasing trend was observed in the EU [2]; meanwhile, the trend in the period from 2017–2021 was stable (neither increase nor decrease in the number of cases was reported) [3]. In 2021, listeriosis was the fifth most commonly reported zoonosis in humans in the EU [3]. It caused 2183 confirmed cases of invasive listeriosis with 923 hospitalisations and 196 deaths [3].

Data regarding the incidence of invasive listeriosis in the EU and around the world suggest that efforts carried out by food business operators, such as the application of the Hazard Analysis and Critical Control Points (HACCP) programme, good agricultural and farming practices, and good hygiene practices are not enough to avoid *L. monocytogenes* risk in RTE foods. Therefore, alternative approaches are necessary to reduce contamination of food and to prevent the growth of *L. monocytogenes* in RTE products. However, in the last years, consumer demands have changed to less processed, more natural foods without chemical additives [4]. Biopreservation and the use of natural enzymes could be attractive options to control *L. monocytogenes* while obtaining clean-label products [5]. Biopreservation is defined as the use of microorganisms and/or their metabolic products to extend the shelf life and enhance the safety of foods [6]. Among the protective cultures that can be used as biopreservative techniques, lactic acid bacteria (LAB) are widely applied. LAB have been involved in food preservation for years due to their capacity to inhibit spoilage and pathogenic bacteria [7] since they produce a wide range of antimicrobial metabolites during growth and food fermentation, such as hydrogen peroxide, lactic acid, acetic acid, low molecular weight substances, antifungal substances, and bacteriocins [8]. In addition, most of them are classified as GRAS (Generally Recognized as Safe) by the FDA (Food and Drug Administration) and as QPS (Qualified Presumption of Safety) by EFSA (European Food Safety Authority). Thus, several LAB cultures are commercialized for food preservation. In addition, some LAB can be considered probiotic bacteria as they have beneficial properties that can improve human and animal health. In this sense, some authors have demonstrated the protective effect of the probiotic bacteria *Lactobacillus rhamnosus* GG against *L. monocytogenes* in fresh-cut fruits [9,10,11]. Among the antimicrobial substances LAB produce, bacteriocins have great potential for use in food processing. They are antimicrobial peptides with bacteriostatic or bactericidal effect against pathogen and spoilage microorganisms [8]. Bacteriocins are considered safe for humans since gastric and pancreatic enzymes present in the stomach during food digestion degrade them into non-toxic substances [12]. Currently, nisin is the only bacteriocin approved by EFSA for its use as a food additive in the EU and accepted by the World Health Organization as a food biopreservative [13,14]. Another strategy to ensure food safety within biopreservation is the use of bacteriophages, which are viruses that infect specific bacteria. They are the most abundant microorganisms on the planet, and they can be found widely in nature. They are considered safe for humans due to their high host-specificity, which means they do not affect human microbiota or microorganisms involved in food production [15]. In 2006, EFSA published a positive scientific opinion about the use of the bacteriophage PhageGuard Listex^TM^ P100 to control *L. monocytogenes* in some RTE foods (meat and poultry, fish and shellfish, and dairy products). However, EFSA highlighted that this bacteriophage should only be used as an additional tool to ensure microbial food safety used in combination with the application of good hygienic practices and good manufacturing practices, and not as a substitute for those practices [16]. Enzymes can also be used as natural preservatives in the food industry. One of the main enzymes used in products such as wine, cheese, sausage, and meat is lysozyme [17], an antimicrobial compound obtained from egg whites and bovine colostrum [18]. Lysozyme hydrolyses β-1,4-glycosidic bonds in the peptidoglycan of bacterial cell walls [19]; therefore, it is more effective against Gram-positive bacteria. In addition, it is not toxic to humans and does not change the physicochemical characteristics of food [17].

Cooked ham is an RTE meat product that can be contaminated with *L. monocytogenes* after thermal treatment; for example, during slicing and packing [20]. In addition, several authors have described the growth of *L. monocytogenes* in sliced and packed cooked ham [21,22]. Fresh cheese is a non-ripened cheese produced by enzymatic coagulation of cow milk with rennet. It is characterized by high moisture content and a high pH, and several authors have demonstrated that *L. monocytogenes* can grow in cheese [23,24]. In addition, a study detected *L. monocytogenes* in 15.4% and 8.9% of environmental samples from artisanal and industrial cheese producers, respectively [25]. *Fuet* is a low-acid dry-cured pork sausage from the Catalonia region (Spain) [26]. During its processing, the product undergoes fermentation that reduces its pH and water activity, increasing the stability of the product [27]. However, the presence and growth of *L. monocytogenes* in low-acidic fermented products has been reported [27,28,29].

In experimental studies conducted at pilot plant scale, *L. monocytogenes* cannot be used because of the risk of cross-contamination in the production of other foods. Therefore, a non-pathogen surrogate organism such as *Listeria innocua* must be used [30,31,32]. In addition, several authors have detected *L. monocytogenes* and *L. innocua* in the same samples from meat and dairy facilities [33,34,35]. Presence of *L. innocua* in food products is also a concern since it is a reservoir of resistance gens which may transfer between bacterial species. Gómez et al. [36] studied the resistance pattern of *Listeria* strains isolated from RTE meat products to various antibiotics. As a conclusion, they observed a multidrug resistance in 24 up to 71 *Listeria* isolates, with a higher prevalence of multidrug resistance in *L. innocua* specie (13.9%) than in *L. monocytogenes* specie (2.9%). In this scenario, control of both species, *L. monocytogenes* and *L. innocua*, could improve microbial food safety. This study aimed to assess the suitability of different commercial biopreservatives, including LAB, the bacteriocin nisin, the bacteriophage Listex^TM^ P100, and the enzyme lysozyme to reduce the population of a *Listeria innocua* cocktail, used as an *L. monocytogenes* surrogate, in three different RTE foods (sliced cooked ham, fresh cheese, and *fuet*). 

## 2. Results

### 2.1. Effect of Antimicrobials on Cooked Ham

A first challenge test was carried out on cooked ham plugs at worst-case conditions (13 °C) to select the most effective techniques. A remarkable *L. innocua* population increase was observed in the control (inoculated only with *L. innocua*) from the initial counts (2.2 ± 0.5 log cfu/g) to 9.0 ± 0.2 log cfu/g after 6 days of storage at 13 °C. At the end of storage (6 days), in four out of seven of the evaluated treatments *L. innocua* population increased considerably in cooked ham plugs (between 5.8 and 6.6 logarithmic units), close to the increase observed in the control treatment (6.8 ± 0.2 logarithmic units). In contrast, *L. innocua* population increased only by 1.3 ± 0.3, 2.2 ± 0.7, and 3.9 ± 0.8 logarithmic units in the treatments with *L. rhamnosus* GG, SafePro^®^ B-SF-43, and PhageGuard Listex^TM^, respectively. Under the same storage conditions (13 °C), a second challenge test was carried out to elucidate if a reduced bacteriophage dose was also effective against *L. innocua*. The results showed that the three studied doses of PhageGuard Listex^TM^ (1, 0.5, and 0.2%) had the same efficacy against *L. innocua* when applied to the surface of cooked ham plugs. Therefore, only the lower doses of bacteriophage were evaluated (0.2 and 0.5%) in the assay carried out at the recommended storage temperature (4 °C and air atmosphere).

**Figure 1:**
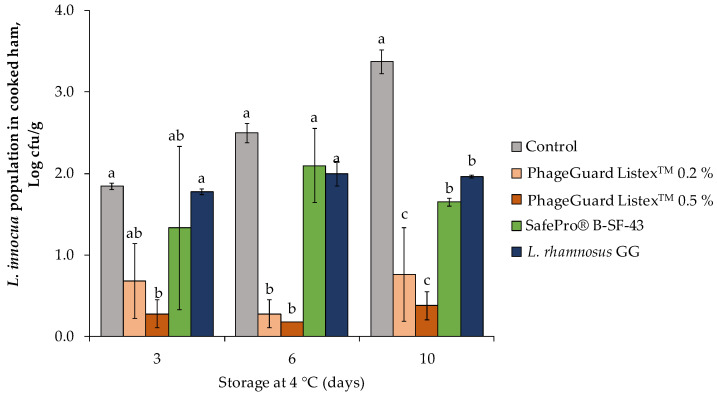
Effect of the biopreservative treatments in *L. innocua* population on cooked ham plugs throughout storage at 4 °C. Values are means ± standard deviations of three biological replicates. Bars marked with different letters (a, b and c) represent statistically significant differences among treatments (*p* < 0.05) at each sampling time.

During the evaluation of the selected biopreservative on cooked ham plugs stored under desirable refrigerated temperature (4 °C), only the application of the highest dose of PhageGuard Listex^TM^ (0.5%) produced a significant reduction in *L. innocua* population after 3 days (Figure 1). In contrast, after 10 days of storage, all the treatments reduced *L. innocua* population and reached counts below 2.0 log cfu/g; meanwhile, in the control counts, it increased up to 3.4 ± 0.1 log cfu/g. The PhageGuard Listex^TM^ at 0.5% reached the highest reduction (3.0 ± 0.2 logarithmic units) followed by the lowest bacteriophage dose (2.6 ± 0.6 logarithmic units), SafePro^®^ B-SF-43 (1.7 ± 0.0 logarithmic reductions), and *L. rhamnosus* GG (1.4 ± 0.0 logarithmic units). 

**Figure 2:**
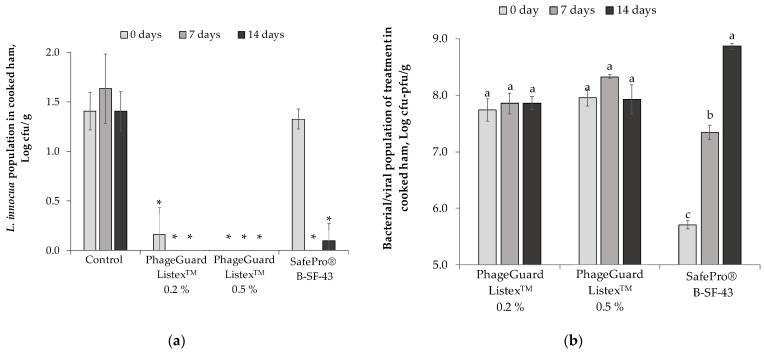
Effect of the biopreservative treatments on sliced cooked ham under semi-commercial conditions (4 °C plus modified atmosphere packaging): (**a**) *L. innocua* population on sliced cooked ham throughout storage. Values are means ± standard deviations of three biological replicates. Bars marked with an asterisk represent statistically significant differences between the treatment and the control (*p* < 0.05) at each sampling time. (**b**) Counts of *Leuconostoc carnosus* (formulated in SafePro^®^ B-SF-43 treatment) and bacteriophage P100 (0.2 and 0.5% PhageGuard Listex^TM^ treatments) on sliced cooked ham throughout storage. Values are means ± standard deviations of three biological replicates. Bars marked with different letters (a, b and c) represent statistically significant differences among sampling time (*p* < 0.05) at each evaluated treatment.

When a semi-commercial evaluation was carried out on cooked ham slices under modified atmosphere packaging (MAP) at 4 °C, an immediate reduction was observed on *L. innocua* counts in the PhageGuard Listex^TM^ treatments after inoculation (Figure 2a). The decrease was higher for cooked ham treated with the highest bacteriophage concentration (0.5%, *L. innocua* population not detected after enrichment) and in the PhageGuard Listex^TM^ 0.2% treatment, in which *L. innocua* decreased below the detection limit (<4 cfu/g). In contrast, *L. innocua* counts in SafePro^®^ B-SF-43 (1.3 ± 0.1 log cfu/g) were not significantly different from the control. Nevertheless, seven days after treatment, SafePro^®^ B-SF-43 reduced *L. innocua* population to no detected bacteria after enrichment as both PhageGuard Listex^TM^ treatments. After 14 days, in the SafePro^®^ B-SF-43 treatment only one of the three trays contained viable cell counts of *L. innocua*, which were below the detection limit (4 cfu/g); meanwhile, the PhageGuard Listex^TM^ treatments (0.2 and 0.5%) reduced *L. innocua* to no detected bacteria after enrichment. 

Regarding the evolution of the biopreservation techniques in treated cooked ham stored at 4 °C under MAP, the counts of *Leuconostoc carnosus* (LAB formulated in SafePro^®^ B-SF-43) increased from 5.7 ± 0.1 log cfu/g to 7.3 ± 0.1 log cfu/g during the first 7 days of storage (Figure 2b) and up to 8.9 ± 0.1 log cfu/g at the end of storage, while bacteriophage levels did not increase during storage compared with initial bacteriophage counts (7.7 ± 0.2 and 8.0 ± 0.1 log pfu/g for 0.2% and 0.5% of PhageGuard Listex^TM^, respectively). Table 1 shows the O_2_ and CO_2_ gas concentration composition of trays measured before sampling. The CO_2_ concentration in trays treated with SafePro^®^ B-SF-43 increased from 25.8 ± 3.4 to 36.6 ± 1.5% after 14 days of storage at 4 °C. Conversely, in the control (*L. innocua* only) and trays treated with PhageGuard Listex^TM^, the CO_2_ concentration decreased with the storage time up to 13.3 ± 0.4% with no significant difference between these treatments. 

### 2.2. Effect of Antimicrobials in Fresh Cheese

**Figure 3:**
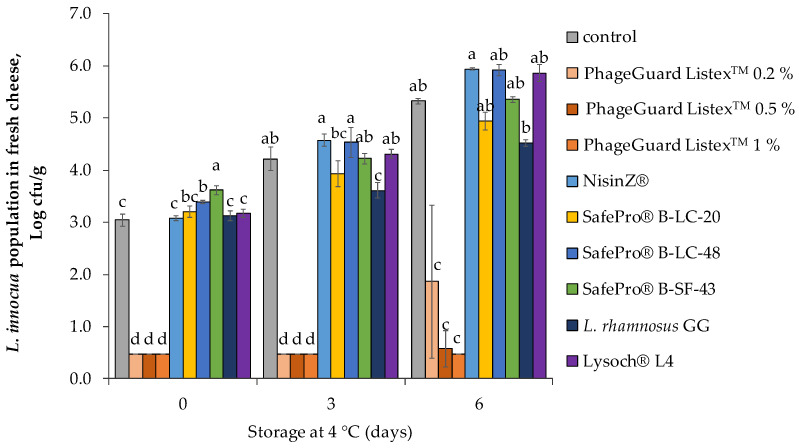
Effect of the biopreservative treatments on *L. innocua* population in fresh cheese throughout storage at 4 °C. Values are means ± standard deviations of three biological replicates. Bars marked with different letters (a, b, c and d) represent statistically significant differences among treatments (*p* < 0.05) at each sampling time.

When the biopreservative agents’ efficacy was evaluated in fresh cheese, antimicrobial agents were introduced directly into the milk used to elaborate the cheese. The effect of the biopreservative treatments applied during the elaboration of fresh cheese is shown in Figure 3. A remarkable *L. innocua* population increase was observed in the control treatment, from the initial population (3.0 ± 0.1 log cfu/g) to 5.3 ± 0.1 log cfu/g after 6 days of storage at 4 °C. Meanwhile, most of the evaluated lactic acid bacteria did not reduce *L. innocua* population, while *L. rhamnosus* GG obtained a slight reduction after 3 days (0.6 ± 0.2 logarithmic units) and 6 days (0.8 ± 0.1 logarithmic units) of storage in front of control. Conversely, all the evaluated doses of PhageGuard Listex^TM^ reduced *L. innocua* population below the detection limit (<10 cfu/g) after inoculation. However, only 0.5 and 1% of PhageGuard Listex^TM^ treatments prevented *L. innocua* regrowth after 6 days of storage. 

**Figure 4:**
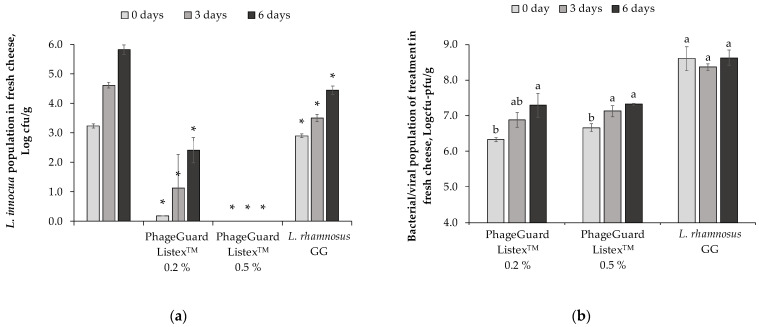
Effect of the biopreservative treatments in fresh cheese under semi-commercial evaluation (4 °C): (**a**) *L. innocua* population in fresh cheese. Values are means ± standard deviations of three biological replicates. Bars marked with an asterisk represent statistically significant differences between the treatment and the control (*p* < 0.05) at each sampling time. (**b**) Counts of bacteriophage P100 (formulated in PhageGuard Listex^TM^ treatment) and *L. rhamnosus* GG in fresh cheese at 0, 3, and 6 days of storage at 4 °C. Values are means ± standard deviations of three biological replicates. Bars marked with different letters (a and b) represent statistically significant differences among sampling time (*p* < 0.05) at each evaluated treatment.

Under semi-commercial conditions, *L. innocua* population increased in the control from 3.2 ± 0.1 log cfu/g to 5.8 ± 0.2 log cfu/g after 6 days in cheese covered with liquid and stored at 4 °C (Figure 4a). After the obtention of the manufactured cheese, a biopreservative effect against *L. innocua* was observed in all the evaluated treatments with reductions higher than 3 logarithmic units in both PhageGuard Listex^TM^ treatments (0.2 and 0.5%) and 0.3 ± 0.1 logarithmic unit reductions in *L. rhamnosus* GG treatment. The decrease of *L. innocua* population in the 0.5% PhageGuard Listex^TM^ treatment was remarkable because it reduced the pathogen population below the detection limit (<10 cfu/g) and no *L. innocua* was detected after enrichment throughout storage. After 6 days of storage, *L. rhamnosus* GG treatment reduced *L. innocua* population up to 4.5 ± 0.1 log cfu/g (1.4 ± 0.1 logarithmic units in front of control) and 0.2% PhageGuard Listex^TM^ obtained reductions of 3.4 ± 0.4 logarithmic units.

In this assay, the bacteriophage P100 concentrations in the obtained fresh cheeses were 6.3 ± 0.1 log pfu/g and 6.7 ± 0.1 log pfu/g in 0.2 and 0.5% bacteriophage treatment, respectively (Figure 4b). Regardless of the initial dose of bacteriophage applied in the milk (7.2 ± 0.1 log pfu/mL and 8.0 ± 0.0 log pfu/mL in treatment with PhageGuard Listex^TM^ 0.2% and 0.5%, respectively), the bacteriophage increased up to 7.3 log pfu/g in fresh cheese. In contrast, the initial population of *L. rhamnosus* GG in the obtained fresh cheese was 8.6 ± 0.3 log cfu/g and levels remained stable throughout storage. 

During the manufacturing of fresh cheese from the semi-commercial assay, the population of *L. innocua* and the biopreservative agents was also determined in the recovered whey after curd separation. The population of *L. innocua* in the recovered whey varied among treatments. Meanwhile, in the control and *L. rhamnosus* GG treatment, counts of 2.0 ± 0.0 and 2.2 ± 0.0 log cfu/mL of *L. innocua* were recovered in the whey; in both treatments with PhageGuard Listex^TM^ the counts of *L. innocua* were below the detection limit (<10 cfu/mL). In addition, all used biopreservants were recovered in the whey with populations of 7.5 ± 0.1 log CFU/mL in *L. rhamnosus* GG treatment, 7.2 ± 0.0 log pfu/mL for 0.5% PhageGuard Listex^TM^, and 6.5 ± 0.1 log pfu/mL for 0.2% PhageGuard Listex^TM^. 

The yield of the obtained cheeses with each biopreservative agent was determined. From 400 mL of inoculated milk, in the control treatment 94.75 g of fresh cheese was obtained, 97.97 g in the *L. rhamnosus* GG treatment, 91.99 g in the PhageGuard Listex^TM^ 0.5% treatment, and 95.25 g in the PhageGuard Listex^TM^ 0.2% treatment. The yield of the produced cheese ranged between 0.24 and 0.25 kg/L of milk.

### 2.3. Effect of Antimicrobials in Fuet

**Figure 5:**
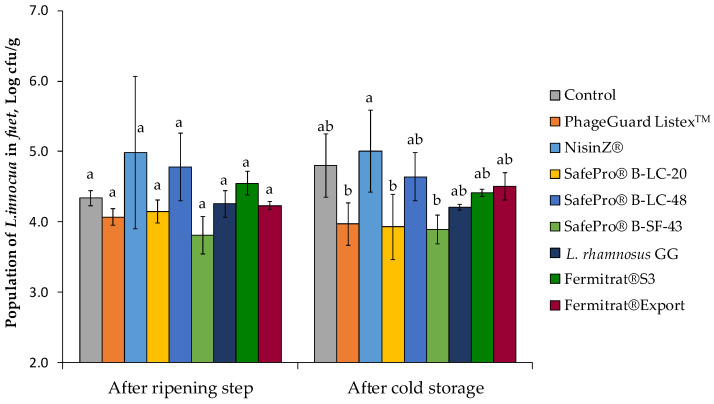
Effect of the biopreservative treatments on *L. innocua* population in *fuet* after ripening (7 days at 15 °C) and cold storage (7 days at 4 °C). Values are means ± standard deviations of three biological replicates. Bars marked with different letters (a and b) represent statistically significant differences among treatments (*p* < 0.05) at each sampling time.

The efficacy of seven biopreservative agents to control *L. innocua* population in *fuet* after ripening and post-cold storage was evaluated. In the *fuet* control (meat inoculated only with *L. innocua*), the pathogen population increased from the initial concentration (4.5 ± 0.6 log cfu/g) to 5.3 ± 0.1 log cfu/g after fermentation (24 h at 22 °C), with an increase of 0.7 ± 0.1 logarithmic units. In contrast, during the ripening process (7 days at 15 °C and 75% R.H.), the population of *L. innocua* in the control treatment reduced by 0.9 ± 0.1 logarithmic units with a final count of 4.3 ± 0.1 log cfu/g (Figure 5). However, the population increased (0.5 ± 0.4 logarithmic units) during post-cold storage, reaching a final population of 4.8 ± 0.4 log cfu/g. In treated samples, SafePro^®^ B-SF-43 obtained the highest population reduction (0.5 ± 0.3 logarithmic reductions) after the ripening step in front of the control. Lower *L. innocua* counts after cold storage were observed in the treatments SafePro^®^ B-SF-43 (3.8 ± 0.3 log cfu/g), SafePro^®^ B-LC-20 (3.9 ± 0.5 log cfu/g), and PhageGuard Listex^TM^ (4.0 ± 0.3 log cfu/g). These three treatments reduced pathogen population around 1 logarithmic unit in front of control at the end of shelf life. 

**Figure 6:**
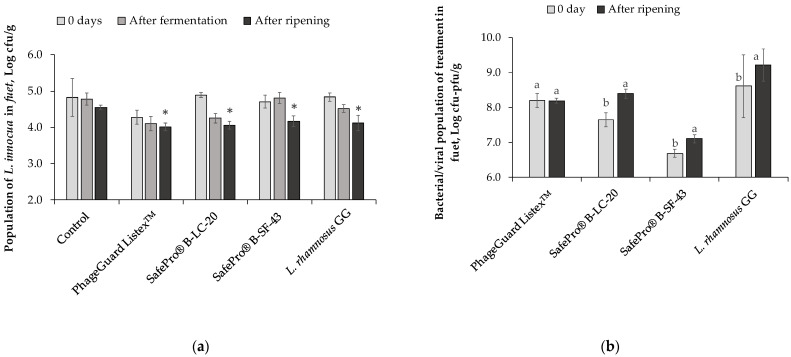
Effect of the biopreservative treatments in *fuet*: (**a**) *L. innocua* population in *fuet* at different time course of *fuet* production: inoculation day (0 day), after 24 h of fermentation, and at the end of ripening (7 days). Values are means ± standard deviations of three biological replicates. Bars marked with an asterisk represent statistically significant differences between the treatment and the control (*p* < 0.05) at each sampling time. (**b**) Counts of bacteriophage P100 (formulated in PhageGuard Listex^TM^ treatment), *P. acidilactici* (formulated in SafePro^®^ B-SF-20 treatment), *Leuc. carnosus* (formulated in SafePro^®^ B-SF-43 treatment), and *L. rhamnosus* GG in *fuet* after inoculation and at the end of ripening. Values are means ± standard deviations of three biological replicates. Bars marked with different letters (a and b) represent statistically significant differences among sampling time (*p* < 0.05) at each evaluated treatment.

The biopreservative treatments that showed the best results were evaluated again against *L. innocua* in *fuet* (Figure 6). In this evaluation, the population of *L. innocua* was enumerated immediately after inoculation (before stuffing in pork casing), after 24 h of fermentation, and at the end of the ripening (Figure 6a). After inoculation, the population of *L. innocua* in the control was 4.8 ± 0.5 log cfu/g, and a slight reduction (0.5 ± 0.2 logarithmic unit) was reached in PhageGuard Listex^TM^ treatment with an *L. innocua* population of 4.3 ± 0.2 log cfu/g. After 24 h of fermentation, no reduction was observed in treatment SafePro^®^ B-SF-43; meanwhile, significant reductions were reached in the treatments PhageGuard Listex^TM^ and SafePro^®^ B-LC-20 with 0.7 ± 0.2 and 0.5 ± 0.1 logarithmic units, respectively, in front of the control. When the *L. innocua* population was enumerated at the end of ripening, all evaluated treatments reached reductions around 0.5 logarithmic units, with the lowest *L. innocua* population recovered in the treatment with 1% of PhageGuard Listex^TM^ (4.0 ± 0.1 log cfu/g). The bacterial or viricidal population of the four evaluated techniques was determined after inoculation (before stuffing) and after ripening (Figure 6b). Whereas bacteriophage population remained stable at the initial inoculated level (8.2 ± 0.1 log pfu/g) until the end of ripening, all lactic acid bacteria populations increased during the ripening process. *Pediococus acidilactici* (formulated in SafePro^®^ B-LC-20) increased 0.8 ± 0.2 logarithmic units to 8.4 ± 0.2 log cfu/g, *Leuc. carnosus* (formulated in SafePro^®^ B-SF-43) increased 0.4 ± 0.2 logarithmic units to 7.1 ± 0.2 log cfu/g, and *L. rhamnosus* GG increased 0.6 ± 0.2 logarithmic units to 9.2 ± 0.2 log cfu/g at the end of *fuet* ripening. 

Table 2 shows the evolution of pH during *fuet* production. In all treatments, pH decreased more than one unit after fermentation (from pH 6.33 ± 0.02, sausage mixture before stuffing). The pH reduction observed in the *L. rhamnosus GG* treatment after fermentation reached the lowest pH value of 5.06 ± 0.04.

## 3. Discussion

This study aimed to compare the efficacy of several biopreservative agents to control a four-strain *L. innocua* cocktail on sliced cooked ham (the evaluated treatments were PhageGuard Listex^TM^, SafePro^®^ B-LC-20, SafePro^®^ B-LC-48, SafePro^®^ B-SF-43, *L. rhamnosus* GG, and NisinZ^®^), fresh cheese (the evaluated treatments were PhageGuard Listex^TM^, SafePro^®^ B-LC-20, SafePro^®^ B-LC-48, SafePro^®^ B-SF-43, *L. rhamnosus* GG, NisinZ^®^, and Lysoch^®^L4), and *fuet* (the evaluated treatments were PhageGuard Listex^TM^, SafePro^®^ B-LC-20, SafePro^®^ B-LC-48, SafePro^®^ B-SF-43, Fermitrat-Export^®^, Fermitrat-S3^®^, *L. rhamnosus* GG, and NisinZ^®^). The most appropriate time to apply the biopreservative agents was selected for each food matrix considering the physicochemical characteristics of the finished food, the microbiota of the raw material, and the manufacturing process steps. Therefore, cooked ham was surface treated after the slicing operation because it could be get contaminated if food manufacturing practices are not applied correctly in the operations that take place after pasteurization or thermal treatment, the last operation aimed to reduce microorganisms. In the case of fresh cheese, the biopreservative agents were added after milk treatment, since *L. monocytogenes* contamination in cheese could be due to survived bacteria to a weak thermal treatment or from cross-contamination after thermal treatment [25,37]. Finally, in *fuet*, the biopreservative agents were also added to the raw materials at the beginning of the production process because no step in the production process has the purpose of reducing microorganism concentration in the raw material. Nevertheless, pH reduction and the reduction in water activity occur in *fuet* during the ripening process could avoid *L. monocytogenes* multiplication. In addition, the introduction of biopreservative agents before a homogenization step allows the spreading of the agents uniformly in food. 

In a previous study, Szczawinski et al. [38] demonstrated the ability of *L. monocytogenes* to grow on contaminated cooked ham at different temperatures (3, 6, 9, 12, and 15 °C), increasing the growth rate with the temperature increase. This study highlights *L. innocua*’s ability to grow on cooked ham plugs stored at 4 °C with an increase of 0.6 logarithmic units after 6 days of storage. However, the pathogen maintained constant population counts when the sliced cooked ham was packaged under modified atmosphere. Furthermore, the *L. innocua* population increased by 2.6 logarithmic units in the fresh cheese after 6 days of storage, although it was packaged in a covering liquid with 2% sodium chloride and stored at 4 °C. In *queso fresco*, which has similar characteristics to fresh cheese, Lourenço et al. [39] observed an *L. monocytogenes* population increase from 3.5 log cfu/g to 7.0 log cfu/g after 11 days of storage at 4 °C, although they inoculated *L. monocytogenes* in the curd instead of the milk. In contrast, the ripening step of the production process of the *fuet* caused a decrease of 0.3 logarithmic units of *L. innocua* after 7 days at 15 °C at 75% humidity. Previously, Porto-Fett et al. [40] validated that *L. monocytogenes* population reduced approximately by 3.6 log cfu/g during the preparation and storage of *fuet*.

To our knowledge, no previous research has compared the efficacy of different biopreservative agents in three food matrices of RTE products with such different characteristics. In this study, on sliced cooked ham, the most effective anti-listerial treatment was PhageGuard Listex^TM^ followed by the SafePro^®^ B-SF-43 agent. Furthermore, PhageGuard Listex^TM^ showed the most effective anti-listerial activity against *L. innocua* in packaged fresh cheese followed by *L. rhamnosus* GG. In both matrices, the evaluated biopreservation techniques reduced *L. innocua* counts by more than 1.5 logarithmic units in front of the control. Conversely, the maximum reduction observed in the manufactured *fuet* was 0.5 logarithmic units with no significant difference among PhageGuard Listex^TM^, SafePro^®^ B-LC-20, SafePro^®^ B-SF-43, and *L. rhamnosus* GG.

The manufacturer’s recommendation for SafePro^®^ B-SF-43 application was 25 g/100 kg of product, which corresponded to 5.7 ± 0.1 log/cfu of *Leuc. carnosum* per gram in our evaluated sliced cooked ham. Budde et al. [41] observed that the rate of inhibition of *L. monocytogenes* in a cooked ham depended on the initial concentration of *Leuc. carnosum* 4010 applied. They reported that when the initial concentration of the *Leuc. carnosum* was 6.3 × 10^6^ cfu/g, *L. monocytogenes* inhibition was observed after 7 days. Meanwhile, when a lower initial concentration (1.2 × 10^5^ cfu/g) was applied the inhibition effect was observed after 14 days. This may be explained by a higher initial lactic acid bacteria population that reached the exponential growth phase earlier. Nevertheless, regardless of the initial *Leuc. carnosum* concentration, at the end of storage (28 days at 5 °C) the cell counts of *L. monocytogenes* were reduced to below 10 cfu/g in both treatments. In this study, *Leuc. carnosum* reached the exponential growth phase after 7 days of storage at 4 °C in the sliced cooked ham. Bacteriocin production by LAB usually starts in this growth phase [42]. In our study, *L. innocua* inhibition was not observed immediately after the application of SafePro^®^ B-SF-43 on the treated slices of cooked ham, and it was delayed to 7 days, when bacteriocins could have been produced, similarly to Budde et al.’s [41] results. The same authors also described the strong anti-listerial activity of *Leuc. carnosum* without producing undesirable flavour in the evaluated RTE meat foods. Although *Leuc. carnosum* established in the *fuet*, it multiplied slightly, around 0.4 logarithmic units after 7 days of ripening. Meanwhile, on the cooked ham, the bacteria increased fourfold compared to the *fuet* (1.6 logarithmic units). The adverse environment (low pH and a_w_, and microbial competition with indigenous fermentation strains) in the *fuet* could have caused the lower *Leuc. carnosum* multiplication and, consequently, lower bacteriocin production than in the cooked ham, in which higher anti-listerial activity was observed. The same anti-listerial rate activity was observed when the SafePro^®^ B-LC-20 was applied in the contaminated mixed meat used to elaborate the *fuet*. *Pediococcus acidilactici* is the bacteria lyophilizate in SafePro^®^ B-LC-20 formulation. Nieto-Lozano et al. [43] evaluated the inhibitory effect of *P. acidilactici* MCH14 against *L. monocytogenes* in Spanish dry-fermented sausage. This pediocin-producing strain reduced the *L. monocytogenes* counts by 2 logarithmic units compared to the control after 30 days of storage. They inoculated the minced meat at a *P. acidilactici* initial concentration of 5 × 10^6^ cfu/g. Although higher initial concentration was applied in our *fuet* (7.7 ± 0.1 log cfu/g), a lower anti-listerial activity was observed. This could be due to the temperature used in our experiment, since a previous study noticed that the activity of *P. acidilactici* was more effective at 4 °C than 15 °C in raw meat [44]. *P. acidilactici* is used as a starter culture for its capacity to reduce food pH; its efficacy as an antimicrobial against *Listeria* has also been confirmed. In addition, Komora et al. [45] and Barbosa et al. [46] concluded that *P. acidilactici* should be considered a potentially useful probiotic. 

Dairy products have extensively been used as food vehicles for *L. rhamnosus GG*, which is the most introduced probiotic strain in food. Rubio et al. [47] confirmed its suitability as a starter culture in cured sausage, and in this study we have evaluated its suitability as an anti-listerial agent in three treated food matrices. The highest *L. innocua* reduction was obtained in the fresh cheese with 1.4 logarithmic units in front of control, as in the cooked ham plugs stored under air conditions a 4 °C, whilst in *fuet* a decrease of 0.5 logarithmic units was achieved at the end of the ripening step. Although the probiotic strain was more effective in the fresh cheese than in the *fuet*, *L. rhamnosus* GG maintained the population counts above 10^8^ cfu/g until the end of storage in both matrices. Therefore, the obtained fresh cheese and *fuet* in this study could be commercialised as functional foods while *L. rhamnosus* GG improves food safety against *L. monocytogenes*. 

Generally, the PhageGuard Listex^TM^ was the most effective biopreservative agent among the evaluated techniques because it showed effective anti-listerial activity in the three evaluated food matrices. In addition, a large reduction of *L. innocua* population was observed in the fresh cheese and the cooked ham. Several authors have also observed anti-listerial activity when applying the bacteriophage P100 in these two food matrices [21,48,49]. Similarly to our results, Holck et al. [21] obtained a 1 logarithmic reduction of *L. monocytogenes* on cooked ham after application of the bacteriophage P100 (5 × 10^7^ pfu/cm^2^) and the surviving pathogen population remained constant until 7 days of storage at 4 °C. Furthermore, higher pathogen reduction after inoculation was observed when we increased the inoculation levels of PhageGuard Listex^TM^ on the sliced cooked ham. The same behaviour tendency was observed in the fresh cheese. Carlton et al. [50] also noticed that the effect of bacteriophages varies with the type of product and is strongly dose dependent. This could be explained by the fact that a high concentration of bacteriophage per unit area is required to ensure interaction with the target pathogen on food surfaces [48]. The lowest initial dose of PhageGuard Listex^TM^ allowed *L. innocua* regrowth in the fresh cheese throughout storage. Regarding bacteriophage multiplication ability, we observed that in the fresh cheese bacteriophage concentration increased with time. The self-replication ability of bacteriophage only occurs if there is still a host present in the food matrix, and the observed biopreservative population increase was in accordance with *L. innocua* survival after the treatment application of PhageGuard Listex^TM^ (0.2%). Therefore, on cooked ham, the P100 population remained stable from inoculation to the end of the evaluation because no *L. innocua* survived after inoculation. Conversely to the lower reduction levels obtained in the *fuet*, Gutiérrez et al. [51] and Komora et al. [45] observed a reduction of 3 logarithmic units of *L. monocytogenes* counts respectively in a Spanish dry-cured ham and in a fermented meat sausage (*Alheira*). However, the addition of extra hurdles, such as high pressure, were necessary to reduce *L. monocytogenes* to below detection limits after 60 days of storage in the fermented sausage. Although an increase of the bacteriophage P100 population was noticed in the fresh cheese when *L. innocua* cells were present, in the *fuet* the bacteriophage population did not increase. Nevertheless, a large quantity of *L. innocua* was present in all evaluated sampling times in the *fuet*. This could be due to the food’s characteristics, such as low moisture and low pH, which did not allow bacteriophage multiplication. In addition, several authors described that in nonliquid food matrices the phage particles appear to become immobilized soon after addition due to limited interaction between the phage and the target bacteria [50,52]. For instance, when Listex^®^ P100 was applied on fresh-cut melon and in melon juice, Oliveira et al. [53] observed the highest effectivity in the liquid matrix. 

Although the results obtained in this study indicate the feasibility of applying biopreservation in the selected food matrices, it is important to highlight this study’s limitations. Firstly, due to the impossibility to work with the human pathogenic *L. monocytogenes*, *L. innocua* was used as a surrogate. However, previously, an in vitro assay was conducted to determine the sensitivity of *L. innocua* selected cocktail in comparison to a cocktail of five strains of *L. monocytogenes* against the different selected biopreservative agents. The results showed that both *Listeria* cocktails presented similar sensibility. In addition, when slight differences in sensibility were observed, *L. monocytogenes* cocktail was more sensitive than the evaluated *L. innocua* cocktail. For this reason, we determined that the selected *L. innocua* strains cocktail was adequate. Secondly, the objective of this study was to evaluate the effect of several biopreservative agents against *L. innocua* in three food matrices susceptible to be linked to listeriosis. Therefore, to quantify *L. innocua* population reduction, the initial levels of *L. innocua* in raw ingredients was above 100 cfu/g, which is a relatively high population. Thirdly, we used a small sample size in this study. However, the aim was to evaluate several options of commercial biopreservatives to obtain knowledge of which could be the best option to food producers. Regardless of the obtained results and the background information, before the application of a new biocontrol tool in a production system, its effectiveness must be evaluated in the specific product considering the food composition, the food physicochemical characteristic, such as pH and water availability, and the production process. Likewise, the evaluated sampling times were approximations of reality, and before biopreservative application in the food chain, longer storage times should be evaluated.

## 4. Material and Methods

### 4.1. Products

The biopreservatives’ efficacy was evaluated in three different food matrices: cooked ham, fresh cheese, and dry-cured Spanish pork sausage (*fuet*). A commercial cooked ham was used to evaluate the anti-listerial efficacy of the biopreservatives. The *fuet* and the fresh cheese were elaborated in the laboratory from the raw materials, pork minced meat and cow pasteurized milk, purchased from a supermarket. Both manufactured foods did not receive any thermal treatment. 

### 4.2. Strains, Commercial Biopreservatives, and Stock Culture Preparation

A cocktail of four strains of *L. innocua* was used in this study as a surrogate of *L. monocytogenes*. These strains were: *L. innocua* CECT 910, *L. innocua* CECT 4030 and *L. innocua* CECT 8848 from *Colección Española de Cultivos Tipo* (CECT), and *L. innocua* TA-1.17 from the Department of Food Technology (UdL) collection. The stock cultures were streaked onto TSA (Tryptone Soy Agar; Biokar, Allone, France) supplemented with yeast extract (6 g/L, Biokar, France; Tryptone Yeast Extract Soy Agar, TYESA) and incubated at 37 °C for 24 h. For each strain, a single colony was picked from the plate and transferred to 10 mL of TSB (Tryptone Soy Broth; Biokar, France) supplemented with yeast extract (6 g/L, Tryptone Yeast Extract Soy Broth, TYESB) and incubated at 37 °C for 20–22 h. The four strains of *L. innocua* were mixed in equal amounts regarding cell numbers to obtain the same concentration of each strain (10^8^ cfu/mL). The cocktail was centrifuged (Sorvall Legend XTR, Thermo Scientific, Waltham, MA, USA) for 10 min at 8900× *g*, and the pellet was resuspended in saline solution (8.5 g/L NaCl, Thermo Fisher, Markham, Canada). For food inoculation, a serial dilution of the *L. innocua* cocktail was prepared in saline peptone solution (SP, 8.5 g/L NaCl and 1 g/L peptone (Biokar, France)) to obtain the desired concentration.

The anti-listerial agents used were the bacteriophage PhageGuard Listex^TM^ (Micreos, Wageningen, the Netherlands), the formulated cultures SafePro^®^ B-LC-20, SafePro^®^ B-LC-48, and SafePro^®^ B-SF-43 (CHR-Hansen, Horsholm, Denmark), and Fermitrat-Export^®^ and Fermitrat-S3^®^ (Amerex, Colmenar Viejo, Spain), the probiotic strain *L. rhamnosus* GG (ATCC 53103), the bacteriocin NisinZ^®^ (Handary, Evere, Belgium), and the enzyme Lysoch^®^L4 (Handary, Evere, Belgium). The evaluated biopreservative agents are currently commercialised products in the EU. According to legislation and manufacturers’ instructions, each biopreservative agent was evaluated in the food matrix in which it is recommended. To apply all biopreservation techniques using the same method, the lyophilised or dehydrated products were suspended in sterile water prior their application. When necessary, the biopreservative agent’s suspension was serially diluted to treat the samples with the same volume of each anti-listerial agent. 

### 4.3. Food Preparation and Application of the Biopreservative Treatments

#### 4.3.1. Cooked Ham

Before removing the packaging of the commercial cooked ham (500 g/unit), its surface was disinfected with ethanol (70%) to avoid cross-contamination. The cooked ham composition was pork (55%), water, potato starch, soy protein, sugar, corn dextrose, stabiliser (E-420 and E-407), preservatives (E-250), antioxidant (E-315), colouring (E-120), and smoked flavour.

Prior to the evaluation of the most suitable techniques on slices of cooked ham in a commercial scenario (4 °C and MAP), two experiments were performed in cooked ham portions under air conditions at different temperatures (13 °C and 4 °C). For these experiments, a slice of cooked ham was cut into plugs of 1.5 cm diameter and 0.5 cm thick (approximately 1 g/plug). Then, 10 µL of the *L. innocua* cocktail was spread over the plug surface to obtain a concentration of 100 cfu/g. After 15 min at room temperature, the plugs were distributed into seven treatments: (1) *L. innocua* control (no treatment), (2) SafePro^®^ B-LC-20 (25 g/100 kg), (3) SafePro^®^ B-LC-48 (12.5 g/100 kg), (4) SafePro^®^ B-SF-43 (25 g/100 kg), (5) *L. rhamnosus* GG (10^8^ cfu/g), (6) NisinZ^®^ (2.5 g/100 kg), and (7) PhageGuard Listex^TM^ (1%). Then, 10 µL of each treatment (previously suspended in water) was spread over the same area. In the *L. innocua* control treatment, 10 µL of sterile water was spread on the ham plugs. The treated inoculated ham plugs were placed in a sterile Petri dish and stored at 13 °C. *L. innocua* population was enumerated after 3 and 6 days of storage. After the initial results evaluation, the most effective techniques were evaluated again on cooked ham plugs stored at 4 °C for 10 days: (1) *L. innocua* control (no treatment), (2) PhageGuard Listex^TM^ (0.2%), (3) PhageGuard Listex^TM^ (0.5%), (4) SafePro^®^ B-SF-43 (25 g/100 kg), and (5) *L. rhamnosus* GG (10^8^ cfu/g). Samples were inoculated as described previously and *L. innocua* population was enumerated after 3, 6, and 10 days at 4 °C.

For commercial evaluation, 216 slices of cooked ham (6 cm diameter and 0.3 cm thick, average 15.0 ± 6.8 g/slice) were inoculated by spraying to obtain a population of 100 cfu/g of *L. innocua*. After 15 min, inoculated slices were distributed in four batches. Each batch was sprayed individually with the selected biopreservative agent (0.1 mL/slice) to obtain the treatments: (1) *L. innocua* control (no treatment), (2) PhageGuard Listex^TM^ (0.2%), (3) PhageGuard Listex^TM^ (0.5%), and (4) SafePro^®^ B-SF-43 (25 g/100 kg). In the *L. innocua* control treatment, sterile water was sprayed onto inoculated slices of cooked ham. For all treatments, six slices (average 89.9 ± 6.8 g/tray) were placed in polypropylene trays (PP, Tecnofood TF013) and sealed with a non-peelable polypropylene film (P12-2050PXNP, Tecnopack, O_2_ permeability of 110 mL/m^2^·24 h·bar at 23 °C) under MAP using a mixture of 30%/70% (CO_2_/N_2_, ALIGAL13, Air Liquid) with a tray sealer machine (ILPRA, Basic, Spain). Nine trays with sliced cooked ham without *L. innocua* were prepared and sealed as packaging control. Sealed trays were stored at 4 °C for 14 days and *L. innocua* and biopreservative agents’ population were enumerated as described below (4.4.) after inoculation and 7 and 14 days of storage. The concentration of O_2_ and CO_2_ in the headspace atmosphere of the trays with MAP was analysed using a handheld gas analyser (CheckPoint, PBI Dansensor, Ringsted, Denmark) before food sampling.

#### 4.3.2. Fresh Cheese

Commercial cow’s low pasteurized milk purchased from a supermarket was warmed at 40 °C. For the first assay, 1.0 L of warm milk was inoculated with 1.0 mL of *L. innocua* cocktail to obtain an initial population of 100 cfu/mL. Then, the inoculated milk was distributed into ten batches of 100 mL. Each batch was individually inoculated with one of the biopreservative agents to obtain ten treatments: (1) *L. innocua* control (no treatment), (2) PhageGuard Listex^TM^ (0.2%), (3) PhageGuard Listex^TM^ (0.5%), (4) PhageGuard Listex^TM^ (1.0%), (5) NisinZ^®^ (1 g/100 kg), (6) SafePro^®^ B-LC-20 (25 g/100 kg), (7) SafePro^®^ B-LC-48 (12.5 g/100 kg), (8) SafePro^®^ B-SF-43 (25 g/100 kg), (9) *L. rhamnosus* GG (10^8^ cfu/g), and (10) Lysoch^®^L4 (16 mL/100 kg). To elaborate fresh cheese, 2 mL/L of calcium chloride (E509, 33%, Laguilhoat, Madrid, Spain) and 0.7 mL/L of rennet (activity of 1 × 10.000, Nievi, Barcelona, Spain) was added to each milk batch (100 mL) and homogenized. Treated milk was incubated at 40 °C for 30 min to promote curd formation. When coagulum was formed, it was cut into 1 cm cubes, stirred, and maintained at 40 °C for 30 min. Whey was drained off using a cotton cheesecloth and curd was transferred into perforated sterile plastic circular cheese containers (5.5 cm in diameter). Samples were stored at 4 °C in a Petri dish and *L. innocua* population was enumerated at initial time and 3 and 6 days of storage.

For the determination of the biopreservation techniques’ efficacy under semi-commercial conditions, 2.0 L of warm milk was inoculated to obtain an initial *L. innocua* population of 100 cfu/mL. Milk was divided into four batches (400 mL) and individually inoculated with the appropriate biopreservative treatment (0.29 mL/100 mL of milk) to obtain: (1) *L. innocua* control (no treatment), (2) PhageGuard Listex^TM^ (0.2%), (3) PhageGuard Listex^TM^ (0.5%), and (4) *L. rhamnosus* GG (10^8^ cfu/g). Then, as described above, 2 mL/L of calcium chloride and 0.7 mL/L of rennet were added to each treatment and milk was incubated at 40 °C for 30 min. Each curd was cut into 1 cm cubes, stirred, and maintained at 40 °C for 30 min. Whey was drained off using a cotton cheesecloth and small half spheres of cheese (2.6 cm diameter) were made using a food grade silicone mould. Nine cheeses (approximately 4.5 g/unit) were obtained from each treatment. Prior to packaging, cheeses were dipped in a salt slurry of 20% sodium chloride for 15 min. Three cheeses of each treatment were introduced to a plastic bag (polyethylene, 14 × 9 cm) with 15.0 mL of covering liquid (containing 2% NaCl) and thermal sealed. Packaged samples were stored at 4 °C for 6 days. *L. innocua* and biopreservative agents’ population were enumerated at initial time and after 3 and 6 days of storage as described below (4.4.). In the semi-commercial assay, the resulting fresh cheeses were weighed, and the yield of each treatment was determined.

#### 4.3.3. Dry-Cured Spanish Pork Sausage (*Fuet*)

For *fuet*, the sausage was prepared with minced pork meat (70:30, lean:fat ratio) and a commercial preparation (Teifel Longaniza 807, marca Teixidor S.L., Spain) was added (52 g/kg). In the first trial, 3.0 kg of sausage mixture was inoculated with 1 mL/100 g of *L. innocua* cocktail to obtain a population of 10^5^ cfu/g. After homogenization, inoculated sausage mixture was divided into nine batches of 300 g. Eight treatments and an *L. innocua* control were evaluated: (1) *L. innocua* control (no treatment), (2) SafePro^®^ B-LC-20 (25 g/100 kg), (3) SafePro^®^ B-LC-48 (12.5 g/100 kg), (4) SafePro^®^ B-SF-43 (25 g/100 kg), (5) *L. rhamnosus* GG (10^8^ cfu/g), (6) Fermitrat-S3^®^ (10 g/100 kg), (7) Fermitrat-Export^®^ (150 g/100 kg), (8) NisinZ^®^ (1 g/100 kg), and (9) PhageGuard Listex^TM^ (1%). In the *L. innocua* control treatment, 1 mL/100 g of sterile water was spread into the inoculated sausage mixture. The same volume (1 mL/100 g) of each biopreservative agent suspension was spread individually and mixed. Each of the treated mixtures were stuffed into natural pork casings (30/32 diameter, packed in salt). Previously, pork casings were conditioned in tap water during 30 min and cleaned. Manufactured *fuets* (approximately 30 g/*fuet*) were placed in a fermentation chamber at 22 °C (saturated humidity) for 24 h to promote fermentation and pH decrease. Ripening of *fuets* took place at 15 °C and 75% relative humidity for 7 days. Prior to biopreservation assays, a trial was realised to determine the time required to achieve a half reduction of the initial weight of the *fuets*. After ripening, the inoculated and treated *fuets* were stored at 4 °C for 7 days. *L. innocua* population was enumerated after ripening and cold storage (7 days at 4 °C). After no observed differences among biopreservative and control treatments in the post-refrigerated storage time of the *fuet*, in the second assay we decided to focus on the production steps and evaluated the action of biopreservative agents only during processing. After the initial results, the most effective techniques were evaluated again at different steps of the *fuet* manufacture process. Treatments were: (1) *L. innocua* control (no treatment), (2) PhageGuard Listex^TM^ (1%), (3) SafePro^®^ B-LC-20 (25 g/100 kg), (4) SafePro^®^ B-SF-43 (25 g/100 kg), and (5) *L. rhamnosus* GG (10^8^ cfu/g). As previously described, 2.0 kg of sausage mixture was inoculated to obtain an initial population of 10^5^ cfu/g of *L. innocua* cocktail. Then, the inoculated sausage mixture was divided into five batches (400 g) and each batch was treated individually with 1 mL/100 g of biopreservation suspension. The control was treated with sterile water. All *fuets* were subjected to a fermentation step (24 h) followed by a ripening of 7 days. *L. innocua* counts were determined after inoculation, after fermentation, and at the end of ripening, and biopreservative agents’ populations were enumerated after their application and at the end of ripening as described below (4.4.), when weight, pH, and water activity (a_w_) were also determined. The pH measurement was made using a handheld pH-meter (Testo 205, Testo SE & Co. KGaA, Titisee-Neustadt, Germany) and the AquaLab (Series 3 TE, Decagon Devices, Inc., Pullman, WA, USA) was used to determine the a_w_. 

### 4.4. Bacterial Enumeration

#### 4.4.1. Food Sampling

To determine *L. innocua* population of the treated cylinders of cooked ham, a plug (approximately 1 g/plug) was placed in a sterile filter plastic bag (80 mL, BagPage^®^ Internscience BagSystem, Saint Nom, France) and diluted with 5 mL of buffered peptone water (BPW, Biokar, Allone, France). Samples were homogenized in a blender for 90 s and *L. innocua* population was enumerated as described below (4.4.2). Each treatment was evaluated in triplicate.

To enumerate pathogen and biopreservative agents’ populations on sliced cooked ham during commercial assay, 3 trays were analysed for treatment at each sampling time. From each tray, 25 g of treated slices in a sterile filter bag (400 mL, BagPage^®^ Internscience BagSystem, France) was homogenised with 75 mL of half Fraser broth (Biokar, France) in a paddle blender for 90 s. One millilitre of the mixture was spread onto PALCAM agar (Biokar, France) by duplicate and incubated at 37 °C for 48 h. Then, the mixture was supplemented with the half Fraser supplement (Biokar, France) and incubated at 30 °C for 24 h. After that, the enriched sample was streaked onto PALCAM, and 0.1 mL was mixed with 10 mL of complete Fraser broth to allow the detection of *L. innocua*. In case of a negative result, enriched tubs were incubated at 30 °C for 24 h and streaked onto PALCAM and COMPASS agar (Biokar, France).

To enumerate the *L. innocua* population in the fresh cheese and in the *fuet*, one gram of sample was placed in a sterile filter plastic bag (80 mL, BagPage^®^ Internscience BagSystem, France) and diluted with 9 mL of BPW. Samples were homogenized in a blender for 90 s and *L. innocua* population was enumerated as described below (Section 4.4.2). Each treatment was evaluated in triplicate. After enumeration, the mixture bags were incubated at 30 °C for 24 h. Then, 0.1 mL of incubated mixture was added to 9 mL of complete Fraser broth when enumeration was below the detection limit. Enriched tubs were incubated, streaked onto PALCAM agar, and plates were incubated at 37 °C for 24–48 h.

#### 4.4.2. *L. innocua* Enumeration

For *L. innocua* enumeration, regardless of the matrices evaluated, the mixture (food homogenized in BPW) was serially diluted in SP, plated onto PALCAM agar, and incubated at 37 °C for 48 h. Plates were counted and results were expressed as log cfu/g.

#### 4.4.3. Biopreservative Agents’ Enumeration

To determine the biopreservative agent counts, the same samples obtained to enumerate *L. innocua* population were used. To enumerate LAB population (treatments: SafePro^®^ B-LC-20 (in *fuet*), SafePro^®^ B-SF-43 (in *fuet* and cooked ham), and *L. rhamnosus* GG (in *fuet* and fresh cheese)), the mixture dilution was plated onto de Man Rogosa and Sharpe agar (MRS, Biokar, France) and incubated at the LAB optimum temperature/time (SafePro^®^ B-SF-43 (*Leuc. carnosum*) at 25 °C for 5 days, and SafePro^®^ B-LC-20 (*P. acidilactici*) and *L. rhamnosus* GG at 37 °C for 24 h). Plates were counted and results were expressed as log cfu/g.

Bacteriophage (P100) counts were determined using the soft agar overlay method following instructions given by the product supplier. Aliquots of 100 µL of the diluted food mixture were mixed with 50 µL of log-phase *L. innocua* (strain 2627 from Micreos collection) in 4.0 mL of Luria–Bertani agar (LB, 10 g/L of Pectic Digest of Meat USP (Biokar, France), 5 g/L of yeast extract, 10 g/L of NaCl, and 4 g/L agar (Condalab, Spain)). Instantly, the soft agar mixture was poured onto solid Brain Heart Infusion agar (BHI, Biokar, France) plates and plates were incubated for 24 h at 30 °C. After incubation, the visible plaques were counted and expressed as log particle forming unit (pfu)/g.

### 4.5. Statistical Analysis

The recovered *L. innocua* counts were transformed into log_10_ to normalize the data. When pathogen detection was positive with an enumeration below the limit of detection (LOD), the count was considered a half of the LOD. In contrast, a negative result after enrichment was considered an absence of *L. innocua*. An analysis of variance (ANOVA) was performed to evaluate the significant effect of each biopreservative treatment at each evaluated time. In the biopreservative agents’ enumeration, counts were transformed into log_10_ to normalize the data. An analysis of variance (ANOVA) was performed to evaluate the significant effect of time at each evaluated treatment. Multiple comparisons were evaluated by Tukey’s Honest Significant Difference (HSD) test with *p* < 0.05 using the JMP Pro 16 software (SAS Institute Inc., Cary, NC, USA). 

## 5. Conclusions

The obtained results have shown that some currently commercialized biopreservation techniques have great potential in reducing the microbiological risk associated with the presence of *L. monocytogenes* in RTE foods. However, all biopreservative agents with the potential to be applied in a food product should be evaluated for each food matrix [49] to select the most efficient method for each product (higher effectivity with lower dose) and to identify the best production step for their introduction. In this sense, it is necessary to consider that the applied LAB cultures do not allow a thermal treatment whilst bacteriophage allows low thermal treatments. Therefore, use of the appropriate biopreservative agent in each RTE product could improve food safety for consumers. However, biopreservation should be used as an extra hurdle against *L. monocytogenes* in combination with the application of current good hygienic practices and good manufacturing practices.

## Figures and Tables

**Table 1:** The O_2_ and CO_2_ concentration in the trays of sliced cooked ham treated with biopreservation techniques and stored at 4 °C under modified atmosphere packaging. Values are means ± standard deviations of three trays.

Treatment	O_2_ (%)			CO_2_ (%)		
	0 Days	7 Days	14 Days	0 Days	7 Days	14 Days
Control	6.1 ± 0.6	8.6 ± 0.1	9.5 ± 0.2	25.8 ± 3.4	15.6 ± 0.8	14.1 ± 0.1
Control *L. innocua*	6.1 ± 0.6	8.2 ± 0.0	9.6 ± 0.0	25.8 ± 3.4	16.1 ± 0.5	13.5 ± 0.6
PhageGuard Listex^TM^ 0.2%	6.1 ± 0.6	8.4 ± 0.1	9.6 ± 0.1	25.8 ± 3.4	16.3 ± 0.3	14.2 ± 0.4
PhageGuard Listex^TM^ 0.5%	6.1 ± 0.6	8.3 ± 0.0	9.7 ± 0.4	25.8 ± 3.4	15.5 ± 0.5	13.3 ± 0.4
SafePro^®^ B-SF-43	6.1 ± 0.6	7.4 ± 0.0	8.4 ± 0.1	25.8 ± 3.4	22.1 ± 1.1 *	36.6 ± 1.5 *

*: The asterisk means statistically significant differences among treatments (*p* < 0.05) at each sampling time.

**Table 2:** Effect of the biopreservation application on pH of *fuet*. Values are means ± standard deviations of three biological replicates. Different letters in the same column show significant differences among treatments (*p* < 0.05) at each sampling time.

pH Value of *Fuet*		
Treatment	After Fermentation	After Ripening
Control *L. innocua*	5.35 ± 0.04 a	5.38 ± 0.04 a
SafePro^®^ B-LC-20	5.19 ± 0.02 b	5.38 ± 0.04 a
SafePro^®^ B-SF-43	5.39 ± 0.02 a	5.44 ± 0.02 a
*L. rhamnosus* GG	5.06 ± 0.04 c	5.06 ± 0.04 b
PhageGuard Listex^TM^	5.42 ± 0.02 a	5.38 ± 0.05 a

## Data Availability

The data presented in this study are available on request from the corresponding author.
